# Fish Oil Increases Diet-Induced Thermogenesis in Mice

**DOI:** 10.3390/md19050278

**Published:** 2021-05-17

**Authors:** Tomomi Yamazaki, Dongyang Li, Reina Ikaga

**Affiliations:** 1 Department of Nutrition and Metabolism, National Institute of Health and Nutrition, National Institutes of Biomedical Innovation, Health and Nutrition, 1-23-1 Toyama, Shinjuku-ku, Tokyo 162-8636, Japan; g1670612@edu.cc.ocha.ac.jp (D.L.); reina017@nibiohn.go.jp (R.I.); 2 The Graduate School of Humanities and Sciences, Ochanomizu University, 2-1-1 Otsuka, Bunkyo-ku, Tokyo 112-8610, Japan

**Keywords:** brown adipose tissue, browning, energy expenditure, n-3 fatty acid, uncoupling protein, white adipose tissue

## Abstract

Increasing energy expenditure (EE) is beneficial for preventing obesity. Diet-induced thermogenesis (DIT) is one of the components of total EE. Therefore, increasing DIT is effective against obesity. We examined how much fish oil (FO) increased DIT by measuring absolute values of DIT in mice. C57BL/6J male mice were given diets of 30 energy% fat consisting of FO or safflower oil plus butter as control oil (Con). After administration for 9 days, respiration in mice was monitored, and then the data were used to calculate DIT and EE. DIT increased significantly by 1.2-fold in the FO-fed mice compared with the Con-fed mice. Body weight gain was significantly lower in the FO-fed mice. FO increased the levels of uncoupling protein 1 (*Ucp1*) mRNA and UCP1 protein in brown adipose tissue (BAT) by 1.5- and 1.2-fold, respectively. In subcutaneous white adipose tissue (subWAT), the levels of *Ucp1* mRNA and UCP1 protein were increased by 6.3- and 2.7-fold, respectively, by FO administration. FO also significantly increased the expression of markers of browning in subWAT such as fibroblast growth factor 21 and cell death-inducing DNA fragmentation factor α-like effector a. Thus, dietary FO seems to increase DIT in mice via the increased expressions of *Ucp1* in BAT and induced browning of subWAT. FO might be a promising dietary fat in the prevention of obesity by upregulation of energy metabolism.

## 1. Introduction

Obesity results when energy intake continuously exceeds energy expenditure (EE). Total daily energy expenditure (TEE) is comprised of multiple components such as basal metabolic rate, diet-induced thermogenesis (DIT) and physical activity-related EE [1]. DIT is defined as an increase in EE above that of the fasting state and is related to digestion, intestinal absorption of nutrients and storage of these nutrients [2]. One of the methods to prevent overweight and obesity is to increase energy consumption by upregulation of DIT [3].

Brown adipose tissue (BAT) is the main site for the induction of DIT and cold-induced thermogenesis, which significantly contributes to controlling body temperature and EE [4]. Although BAT is considered to be abundant in small rodents and human infants and decreases with aging in human [5], recent studies showed that functional BAT was identified in adult human [6,7]. The thermogenic ability of BAT is principally dependent on uncoupling protein 1 (UCP1) [8,9]. UCP1 facilitates uncoupling of mitochondrial substrate oxidation from ATP production, which leads to energy release as heat from free fatty acid oxidation [4].

UCP1-ablated mice consumed less oxygen than wild-type mice during the eating period, that is, DIT was UCP1-dependent [10]. UCP1-deficient mice maintained in a room at 23 °C developed obesity with age; therefore, UCP1 may play an important role against obesity [11]. UCP1 gene polymorphism (−3826 A/G) showed lowered capacity of thermic effect in response to dietary intake in healthy boys aged 8–11 years [12]. Thus, the function of UCP1 and activity promoting the activation of BAT greatly contribute to the increase of DIT.

BAT is strongly activated by exposure to cold and by pharmacological effects, such as that of β3-adrenergic receptor agonist [6,13,14]. Moreover, it has been reported that BAT is activated by food ingredients such as capsinoids, thereby contributing to a reduction in body fat [15,16]. Fish oil (FO) also has anti-obesity effects in humans [17,18,19]. FO contains a high content of n-3 polyunsaturated fatty acids, eicosapentaenoic acid (EPA) and docosahexaenoic acid (DHA), which must be obtained from the diet or synthesized from alpha-linolenic acid in the body [20,21,22]. DHA and EPA bind to peroxisome proliferator-activated receptor (PPAR) α and thereby activate PPARα [23,24], which is highly expressed in BAT [25]. PPARα binds to the PPAR response element of the *Ucp1* gene to increase mRNA expression of *Ucp1* [26].

Recently, beige adipose tissue, which is produced by the browning of white adipose tissue (WAT), has been reported as a third type of adipose tissue in addition to WAT and BAT [27,28,29]. Beige adipocytes are strongly induced by some environmental conditions and external cues such as exposure to cold and some pharmacological treatments, and they have potent thermogenic ability similar to that of classical brown adipocytes [30]. FO treatment leads to the browning of WAT, increases thermogenic genes such as *Ucp1* [31,32,33], stimulates thermogenesis, as measured by rectal temperature [34,35], and increases EE without changes in food intake [36]. These studies only suggest the possibility that FO influences DIT, however, and how much FO actually increases DIT is still unknown.

We recently developed a new technique to measure absolute DIT values in mice by applying a methodology used in the measurement of DIT in human to mice using a respiratory chamber [37]. In the present study, we showed how much FO increased DIT through activation of BAT and browning of WAT. An increase in DIT may have potential impact on anti-obesity and therapy for diabetes [6,7,38], and the evidence shown in this study indicates that FO might be a promising dietary fat.

## 2. Results

### 2.1. Effects of Fish Oil Supplementation on DIT, EE, Activity and RER

**Figure 1:**
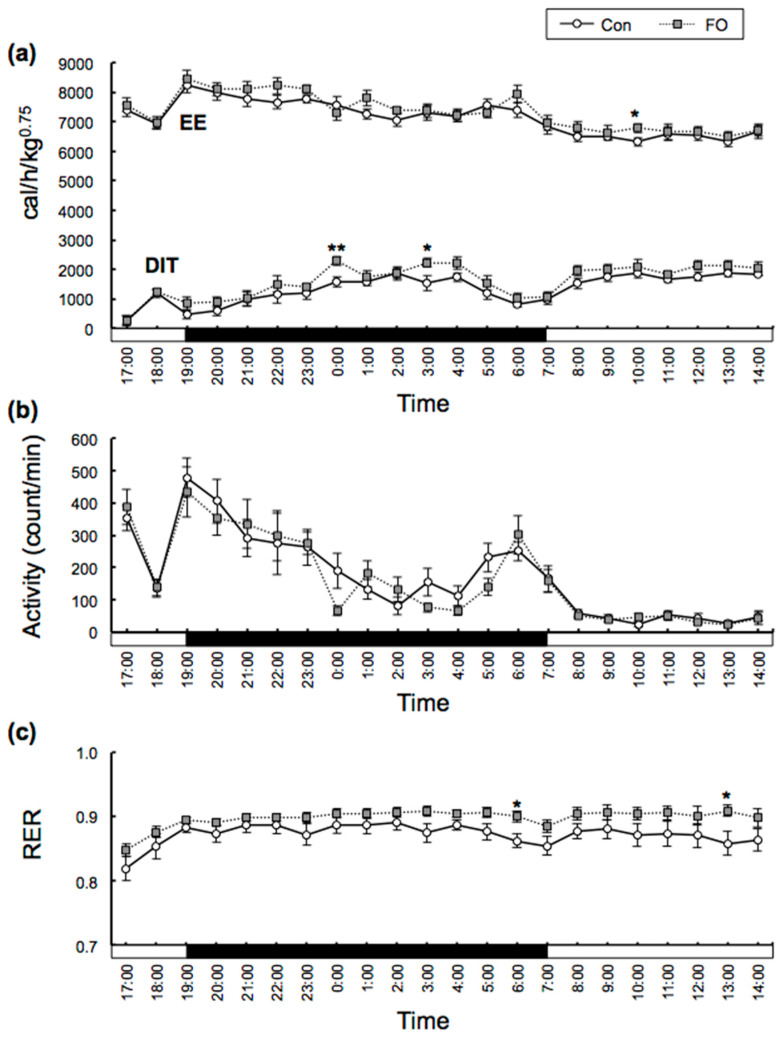
Time course of diet-induced thermogenesis (DIT), energy expenditure (EE), activity and respiratory exchange ratio (RER) in the control fat (Con)- and fish oil (FO)-fed male mice. The measurements were carried out over a 22-h period. The data of EE (upper lines), DIT (lower lines) (**a**), activity (**b**) and RER (**c**) are shown for every hour. White circles and gray squares represent data from the Con- and the FO-fed mice, respectively. The black and white bars on the x axis represent dark and light cycles, respectively. Values are mean ± SEM (n = 7). * *p* < 0.05, ** *p* < 0.01 vs. Con-fed mice. Significant differences between two groups were tested by Student *t*-test.

**Figure 2:**
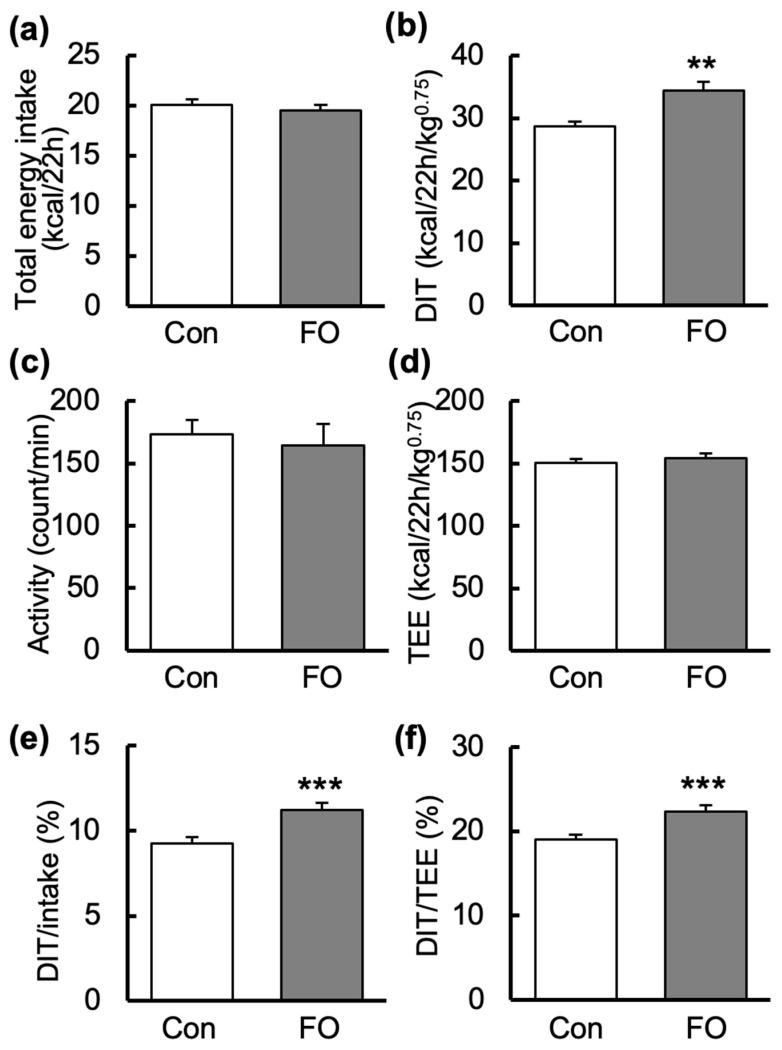
Values of total energy intake, diet-induced thermogenesis (DIT), activity and total energy expenditure (TEE) during DIT measurement in the control fat (Con)- and fish oil (FO)-fed male mice. Total energy intake (**a**) at measurement of energy metabolism was estimated by subtracting the food weight at the completion of measurement from the initial food weight measurement. The values of total DIT (**b**), activity (**c**) and TEE (**d**) were calculated from measurements taken over 22 h under the fed condition. DIT/intake (**e**) and DIT/TEE (**f**) were calculated by dividing total DIT by total calorie intake and by TEE, respectively. White and gray columns represent data from the Con- and FO-fed mice, respectively. Values are mean ± SEM (n = 7). ** *p* < 0.01, *** *p* < 0.001 vs. Con-fed mice. Significant differences between two groups were tested by Student *t*-test.

Energy metabolism of mice was measured after 9 days of feeding of each experimental diet. The measurements of O_2_ consumption, CO_2_ production and activity (defined as the count per minute of any movement made by mouse) of the mice were carried out over a 22-h period. The DIT of the control fat (Con)-fed mice began to increase as soon as they started eating, was maintained at a high level during the dark period, and then decreased toward the end of the dark period (Figure 1a). However, DIT increased again after the start of the light period. Similar changes were observed in the FO-fed mice (Figure 1a). When comparing the DIT in the Con- and FO-fed mice every hour, DIT in the FO-fed mice was significantly higher at 0000 and 0300. EE in the dark period and light period was not different in the Con- and FO-fed mice (Dark: Con, 7615 ± 76 cal/h/kg^0.75^; FO, 7582 ± 87 cal/h/kg^0.75^; Light: Con, 6712 ± 83 cal/h/kg^0.75^; FO, 6641 ± 92 cal/h/kg^0.75^). However, DIT in the dark period was higher in the FO-fed mice than that in the Con-fed mice (Dark: Con, 1275 ± 22 cal/h/kg^0.75^; FO, 1541 ± 32 cal/h/kg^0.75^, *p* < 0.01; Light: Con, 1509 ± 47 cal/h/kg^0.75^; FO, 1674 ± 46 cal/h/kg^0.75^). There was no difference in activity every hour between the two groups (Figure 1b). Activity in the dark period and light period was not different in the Con- and FO-fed mice (Dark: Con, 238.2 ± 13.2 count/min; FO, 221.2 ± 23.4 count/min; Light: Con, 95.4 ± 5.8 count/min; FO, 96.5 ± 11.7 count/min). The respiratory exchange ratio (RER) in the FO-fed mice was higher at 0600 and 1300 than that in the Con-fed mice, but there was no significant difference at other times (Figure 1c). RER in the dark period and light period was not different in the Con- and FO-fed mice (Dark: Con, 0.881 ± 0.010; FO, 0.901 ± 0.006; Light: Con, 0.860 ± 0.005; FO, 0.893 ± 0.009). The total energy intake during DIT measurement was almost the same in the Con- and the FO-fed mice (Figure 2a). Total DIT over 22 h was calculated from the area under each curve. Total DIT in the FO-fed mice was 1.2-fold higher than that in the Con-fed mice (Figure 2b). TEE over 22 h was also calculated from the area under each curve. The values of activity and TEE between the two groups were not different (Figure 2c,d). DIT (%) versus calorie intake was calculated by dividing total DIT by total calorie intake and is indicated as DIT_/intake_ in Figure 2e. DIT_/intake_ was 11.2% for the FO-fed mice, which was 1.2-fold higher than that for the Con-fed mice. DIT (%) versus TEE was calculated by dividing total DIT by TEE and is indicated as DIT_/TEE_ in Figure 2f. DIT_/TEE_ for the FO-fed mice was 22.3%, which was also 1.2-fold higher than that for the Con-fed mice.

### 2.2. Body Weight and Tissue Weights of Con- and FO-Fed Mice

The mean energy intake was similar between the Con- and the FO-fed mice during the 10-day administration period (Con, 17.6 ± 0.8 kcal/day; FO, 17.7 ± 0.6 kcal/day, *p* = 0.97). Although final body weight (BW) was not different between the Con- and FO-fed mice, the BW gain in the FO-fed mice was significantly lower than that in the Con-fed mice during the 10-day period (Con, 10.3 ± 1.4%; FO, 6.6 ± 1.0%, *p* < 0.05). The weights of subcutaneous WAT (subWAT) in the FO-fed mice were not different from those in the FO-fed mice (*p* = 0.07, Table 1). However, the weights of epididymal WAT and mesenteric WAT in the FO-fed mice were lower than those in the Con-fed mice. The weight of BAT was not affected by FO supplementation.

### 2.3. Serum Chemicals of Con- and FO-Fed Mice

Because the weights of the epididymal WAT and mesenteric WAT in the FO-fed mice were lower than those in the Con-fed mice, we analyzed serum concentrations of glucose, non-esterified fatty acid (NEFA), triglyceride (TG) and total cholesterol (TC). The concentrations of serum glucose in the Con- and the FO-fed mice were the same (Table 2). However, the serum concentrations of NEFA, TG and TC in the FO-fed mice were significantly lower than those in the Con-fed mice (Table 2).

### 2.4. Effects of FO Supplementation on BAT

**Figure 3:**
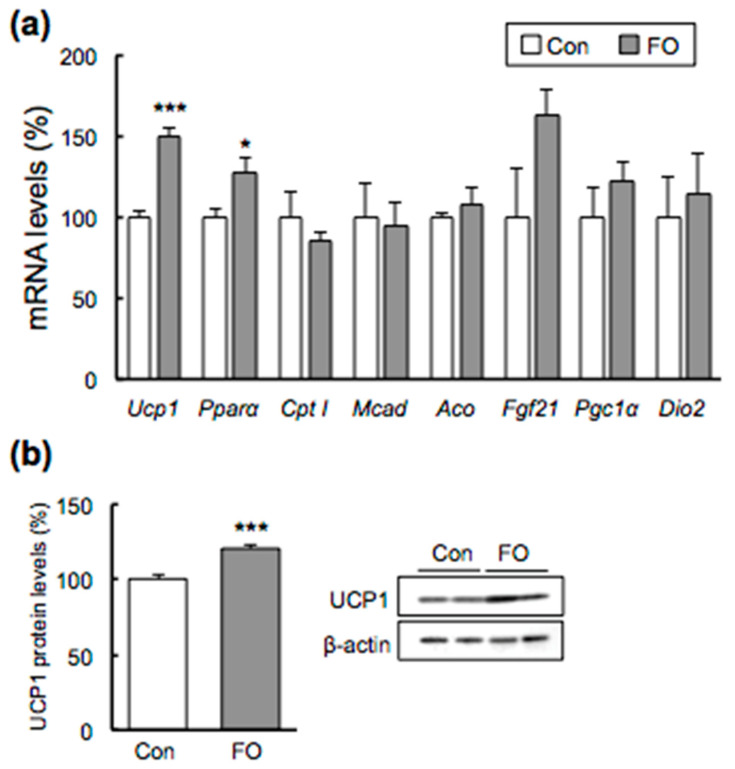
Effect of fish oil (FO) supplementation on gene expression and UCP1 protein levels in brown adipose tissue. mRNA levels of *Ucp1* and *Pparα* and its target genes (**a**) and UCP1 protein (**b**) were assessed by quantitative RT-PCR or western blotting. β-actin was used as the normalization control. The percent of mRNA and protein levels relative to those of Con-fed mice are indicated. White and gray columns represent data from the Con- and FO-fed mice, respectively. Values are mean ± SEM (n = 7). * *p* < 0.05, *** *p* < 0.001 vs. Con-fed mice. Significant differences between two groups were tested by Student *t*-test.

To confirm the mechanism of increase of DIT in the FO-fed mice, we examined expression profiling of the *Ucp1* gene and UCP1 protein. FO supplementation resulted in a 1.5-fold increase in *Ucp1* mRNA in BAT (Figure 3a). UCP1 protein expression was also analyzed (n = 7 in each group), and representative data (n = 2 in each group) indicating a 1.2-fold increase in expression are shown in Figure 3b. The mRNA expression of *Pparα*, which is one of the nuclear transcription factors whose activation leads to increased fatty acid β-oxidation [39], was significantly increased by FO supplementation (Figure 3a). However, FO supplementation did not affect the mRNA expressions of target genes carnitine palmitoyltransferase I (*Cpt I*), acyl-CoA oxidase (*Aco*) and medium-chain acyl-CoA dehydrogenase (*Mcad*) (Figure 3a). Fibroblast growth factor 21 (*Fgf21*) expression was also not increased by FO administration (Figure 3a). The expression of the mitochondria biogenesis marker peroxisome proliferator-activated receptor gamma coactivator 1-alpha (PGC1α) and that of crucial thermogenesis biomarker type 2 iodothyronine deiodinase (Dio2) in the mice was not different between the two groups (Figure 3a). No difference in β3-adrenergic receptor (β3-AR) mRNA was observed in BAT (Con, 100.0 ± 7.0%; FO, 93.0 ± 11.4%).

### 2.5. Effects of FO Supplementation on Gene Expression in subWAT

**Figure 4:**
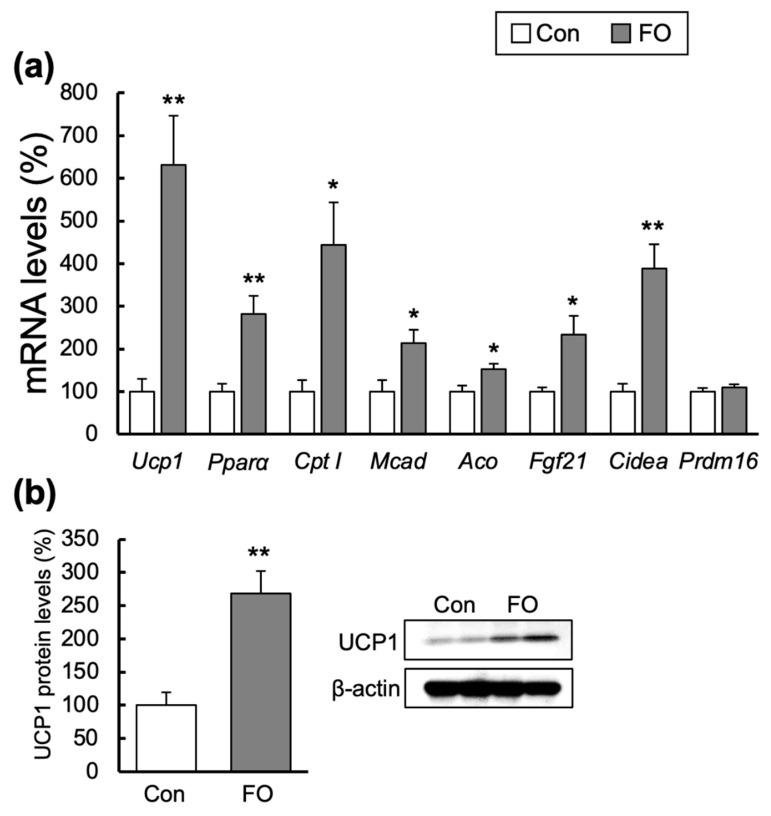
Effect of fish oil (FO) supplementation on gene expression and UCP1 protein levels in subcutaneous white adipose tissue. The mRNA levels of *Ucp1*, *Pparα* and its target genes and beige adipocyte-specific gene (**a**) and UCP1 protein (**b**) were assessed by quantitative RT-PCR or western blotting. β-actin was used as the normalization control. The percent of mRNA and protein levels relative to those of control fat (Con)-fed mice are indicated. White and gray columns represent data from the Con- and the FO-fed mice, respectively. Values are mean ± SEM (n = 7). * *p* < 0.05, ** *p* < 0.01 vs. Con-fed mice. Significant differences between two groups were tested by Student *t*-test.

FO dramatically increased *Ucp1* mRNA expression by 6.3-fold in the subWAT (Figure 4a). UCP1 protein levels were also analyzed (n = 7 in each group), and representative data (n = 2 in each group) indicating a 2.7-fold increase compared with those in the Con-fed mice are shown in Figure 4b. FO supplementation also caused higher expressions of *Pparα* and its target genes of *Cpt I*, *Aco* and *Mcad* in comparison to those in the Con-fed mice (Figure 4a). Fgf21 expression in the FO-fed mice was also increased by 2.3-fold compared with that in the Con-fed mice (Figure 4a). β3-AR mRNA expression was higher in subWAT from the FO-fed mice than that in the Con-fed mice (Con, 100.0 ± 17.9%; FO, 246.1 ± 40.4%, *p* < 0.001).

Among the brown fat-selective genes, expression of cell death-inducing DNA fragmentation factor α-like effector a (Cidea) was significantly increased by 3.9-fold in the FO-fed mice compared with that in the Con-fed mice, whereas that of PR domain containing 16 (Prdm16) mRNA was not different (Figure 4a).

### 2.6. Effects of FO Supplementation on Gene Expression in Liver

As the effects of supplementation could also be caused by increased metabolism in the liver, we analyzed gene expressions in the liver related to fatty acid β-oxidation and fatty acid synthesis. As shown in Table S1, fatty acid β-oxidation was induced the FO-fed mice, and fatty acid synthesis was decreased.

## 3. Discussion

We found that FO increased DIT in mice by 1.2-fold along with the activation of BAT caused by the increased expression of UCP1 and the browning of subWAT. As females are reported to produce less heat than males, we used male mice for our experiment [40]. We observed DIT in both the light period and dark period, although it was higher in the latter because mice eat principally in the dark period. Actually, the Con and FO groups of mice took about 70–80% and 20–30% of their total food intake in the dark and light periods, respectively. It appeared that maintenance of DIT in the light period was caused by feeding in the light period (Figure 1a).

In this study, FO increased the expression of the *Ucp1* gene in BAT by 1.5-fold. Other researchers also reported that FO administration both in vitro and in vivo induced the increased expression of *Ucp1* in BAT. *Ucp1* mRNA expression and UCP1 protein levels were both significantly increased in brown progenitor cells isolated from interscapular BAT supplemented with EPA [35]. EPA also increased mitochondrial content in a dose-dependent manner in HIB 1B brown adipose cells [41]. EPA administration to C57BL/6J mice for 11 weeks, and DHA-enriched FO (DHA 25%, EPA 8%) or EPA-enriched FO (DHA 12%, EPA 28%) administration to mice for 10 weeks, significantly increased UCP1 protein levels and *Ucp1* mRNA expression in BAT [34,42]. UCP1 activity in BAT was significantly increased in rats fed with EPA or a mixture of EPA and DHA for 4 weeks by GDP binding [43]. These reports support our results that UCP1 expression was significantly increased by FO administration in BAT (Figure 3a,b). The nuclear receptor PPARα regulates the expression of *Cpt I*, *Mcad* and *Aco*, which are involved in the fatty acid β-oxidation [41,44,45]. FO administration increased the expression of *Pparα* mRNA by 1.3-fold (*p* < 0.05), but expressions of its target genes *Cpt I*, *Mcad* and *Aco* were not affected in BAT, although that of the other Pparα target gene, *Ucp1*, increased (Figure 3a). Kim et al. reported that the expression of *Cpt I* mRNA in BAT of mice fed EPA-enriched FO increased significantly compared with that of control mice. In contrast, the expression of *Cpt I* mRNA did not increase in mice fed DHA-enriched FO, which has a similar fatty acid ratio as in the present study [34]. The reason why EPA-enriched FO could, but DHA-enriched FO could not, induce *Cpt I* expression in BAT is currently not clear. Further study will be required to reveal the mechanism.

FO also increased the expressions of the *Ucp1* gene and other genes related to the fatty acid β-oxidation in subWAT (Figure 4a). In terms of the marker of browning, *Cidea* mRNA was increased by 3.9-fold, but the expression of *Prdm16* was not increased (Figure 4a). FO enhances fatty acid oxidation through PPARα activation in WAT and causes browning of subWAT [34,46]. When cells derived from subcutaneous adipocytes from overweight females were treated with 200 μM EPA, expressions of *UCP1* and *CIDEA* mRNA increased significantly. The mRNA expression of *PRMD16* increased significantly with 100 μM EPA treatment but not with 200 μM EPA treatment [31]. When the stromal vascular cells isolated from subWAT of C57BL/6J mice were treated with 200 μM EPA during a differentiated process, the expressions of fatty acid β-oxidation-related genes *Ucp1*, *2*, *3* and *Cpt I*, and *Cidea*, increased significantly, but that of *Prdm16* was still not increased as in our results [32]. The reasons for FO causing different expressions of *Cidea* and *Prdm16* are currently unknown. Due to the increased expressions of *Ucp1* and *Cpt I* mRNA in FO-fed mice, the brown adipocyte-like phenotype was induced in subWAT [33]. The PPARα agonist is known to promote browning in subWAT [47,48] and increase the body temperature [48]. Contrary to these reports, UCP1 protein is reported to be very low or undetectable in subWAT even though mice were fed FO [42]. Our results supported the findings that FO administration markedly increased UCP1 expression in subWAT and induced subWAT browning. Beige adipocytes were shown to have potent thermogenic ability comparable to classical BAT [30], and the thermogenic density and total quantitative contribution in subWAT were maximally one-fifth and one-third of all BAT mitochondria, respectively [49]. Thus, the classical BAT depots would still be predominate in thermogenesis, but the browning of WAT would also contribute to thermogenesis. Sato et al. recently showed that phospholipase A2 group IID, which is expressed in M2-type macrophages in WAT, released n-3 fatty acid and increased energy expenditure and rectal temperature by facilitating subWAT browning, which ameliorated diet-induced obesity [50]. Thus, FO-caused browning of WAT might also contribute to inducing DIT.

FGF21 is reported to have an endocrinological role in BAT and WAT [51,52]. The expression of *Fgf21* mRNA in subWAT increased dramatically in mice after exposure to cold [52]. Moreover, the differentiated primary subWAT treated with β-agonist synthesized and secreted FGF21, suggesting that adipose FGF21 may act mainly in a paracrine/autocrine manner [52]. However, similar to the previous research concerning FO [34], the expression of *Fgf21* did not increase with FO administration in BAT (Figure 3a). However, contrary to that report, *Fgf21* expression in the present study was significantly increased by FO administration in subWAT (Figure 4a). This result leads us to the hypothesis that increased *Fgf21* of subWAT might induce the browning of WAT observed in the present study.

FO is reported to induce UCP1 expression in BAT and WAT via the sympathetic nervous system and transient receptor potential vanilloid 1 [34]. In the present study, β3-AR mRNA expression was higher in subWAT from the FO-fed mice than that in the Con-fed mice. However, no difference in β3-AR mRNA was observed in BAT. Although we did not determine the direct influence of fish oil on sympathetic flow, over the short term of 10 days, FO intake might induce UCP1 expression in subWAT via the sympathetic nervous system at least in part.

G-protein-coupled receptor 120 (GPR120), a receptor for n-3 polyunsaturated fatty acids, was also suggested to contribute to thermogenic activation in BAT and WAT by n-3 fatty acids by suppressing tissue inflammation induced by macrophages, especially in obese mice [53,54,55,56]. We used non-obese mice, and the expression of *Gpr120* was not affected in BAT and subWAT by FO supplementation (data not shown).

A systematic review indicated that EPA and DHA lowered serum lipid levels such as TG concentration [57]. Some mechanisms of serum lipid lowering by FO have been reported. EPA increased lipid oxidation in rat liver and reduced serum lipids [58]. FO also lowered serum lipids in adult human subjects [59]. We previously reported that FO administration at the same dose as in the present study decreased fatty acid synthesis genes such as acetyl-CoA carboxylase and increased fatty acid oxidation genes such as *Cpt I*, *Mcad* and *Aco* in mouse liver [60]. The rate limiting step in mitochondrial fatty acid oxidation is mediated by CPT I [61]. Even though CPT I activity in WAT was still low compared with that in liver and BAT in rat [62], activation of CPT I by overexpression of CPT I in 3T3-L1 adipocytes reduced NEFA release [63]. These FO-induced mechanisms in liver and WAT may contribute to lowering of the serum lipid levels. In general, enhanced fatty acid oxidation in the whole body is related to decreased RER. However, in human, RER correlated negatively with plasma palmitate concentrations [64]. In the present study, FO administration caused decreased serum concentrations of NEFA and TG (Table 2). We showed here that the RER of the FO-fed mice was slightly higher than that of the Con-fed mice (Figure 1c), although not significantly so. This was probably due to the reduced serum lipid levels in the FO-fed mice.

The short period of FO administration of 10 days in the present study did not result in weight loss, but weight gain and the weights of epididymal and mesenteric WAT were significantly reduced. Mice fed 21.42 or 42.84 energy% (en%) FO for 6 weeks significantly reduced BW by about 1.5 g or 4 g, respectively [36]. It is likely that mice need to be fed FO for a longer period of time to reduce their weight. BAT-positive subjects would undergo higher DIT than BAT-negative subjects [65]. Thus, BAT activation is expected to have an anti-obesity effect. Interestingly, BAT-positive subjects (young healthy men) showed an increase in EE after oral ingestion of capsinoids (9 mg) [15]. Moreover, capsinoids 6 mg/day taken orally for 12 weeks promoted loss of human abdominal fat [66]. FO supplementation in the present study resulted in a 1.2-fold increase in DIT_/intake_ (Figure 2e). In human, DIT uses 10% of the daily energy intake [67]. The estimated energy requirement for adult men is about 2500 kcal/day [68], and the energy consumed by DIT was calculated to be about 250 kcal/day, and 300 kcal/day if multiplied by 1.2. Thus, a 1.2-fold increase in DIT was estimated to maximally increase energy consumption by 50 kcal/day. Adult human adipose tissue contains 71.6% crude fat [69]. Therefore, an increase in DIT by 1.2-fold was estimated to indicate fat burning of 500 g of adipocytes over about 2 months.

In conclusion, we first showed that FO supplementation significantly increased DIT by 1.2-fold. DIT_/intake_ and DIT_/TEE_ for the FO-fed mice were 11.2% and 22.3%, respectively. The FO-increased DIT was complemented by the increased expression of UCP1, activation of BAT and subWAT browning. FO may be a promising dietary fat for the prevention of overweight and obesity.

## 4. Materials and Methods

### 4.1. Animals

Seven-week-old male C57BL/6J mice were obtained from Tokyo Laboratory Animal Science (Tokyo, Japan). They were fed a standard laboratory diet (CE2) from CLEA Japan, Inc, (Tokyo, Japan) for 1 week for stabilization of their metabolism. Mice were maintained under a controlled environment at 22 °C in a 12-h light (0700–1900 h)/12-h dark (1900–0700 h) cycle. They were housed individually and allowed access to the experimental diets and water ad libitum. Care of the mice followed guidelines of the National Institutes of Health’s Guide for the Care and Use of Laboratory Animals. The National Institutes of Biomedical Innovation, Health and Nutrition, Japan, reviewed and approved all animal procedures (Approval no. DS27-52R3).

### 4.2. Diet

Mice received a fat-rich diet (30 en%) containing either mixed fat with safflower oil and butter (control) or FO (n = 7 in each group). Diets were prepared as described previously [60,70], and the composition of the diet is listed in Table 3. Butter and safflower oil were purchased from Snow Brand Milk Corp. (Hokkaido, Japan) and Benibana Food (Tokyo, Japan), respectively. FO (containing 7% EPA and 24% DHA) was kindly provided by the NOF Corporation (Tokyo, Japan). The food was provided to the mice every day. To estimate daily food intake, the food weight of each day was subtracted from the initial food weight of the previous day. Mean food intake over the entire experimental period in the two groups of mice was calculated using these data. The diets were offered for 10 days.

### 4.3. Measurement of O_2_ Consumption and CO_2_ Production to Calculate DIT and Energy Production

Mice on the 9th day of the experimental diet administration were used for the experiment. The method for calculating DIT was described previously [37]. Briefly, mice were placed in the calorimeter without food 6 days before starting the experiment at 1700 h, and then energy metabolism was measured for the 11-h period from 0000–1100 h. Oxygen consumption (VO_2_) and carbon dioxide production (VCO_2_) were monitored with a system that measures O_2_/CO_2_ metabolism in small animals (MK-5000RQ; Muromachi Kikai Co., Ltd., Tokyo, Japan), and their values were used to calculate DIT and EE. The EE was calculated as follows: EE (kcal/min) = 3.9 VO_2_ + 1.1 VCO_2_ [71]. For the measurements made after feeding, the same mice used in the fasted measurements were placed in the calorimeter at 1600 h. The research diet was provided at 1700 h, and energy metabolism was measured over the 22-h period from 1700–1500 h. VO_2_, VCO_2_ and activity were monitored by the system at 3-min intervals, and every four data points were averaged. The average value over the 12-min period was considered the mean value. The data were normalized to the square root of the activity count. Under the fasting conditions, 55 (5/h × 11 h) values each for EE and activity were selected from the measurements obtained over the 11-h period; we then plotted EE against the square root of activity and identified a linear regression equation by simple linear regression analysis. Under the fed conditions, 110 (5/h × 22 h) values each for EE and activity were selected from the measurements obtained over the 22-h period, and EE was then plotted against the square root of activity.

### 4.4. Quantitative Real-Time PCR

On the 10th day of the experimental diet, mice were sacrificed by cervical dislocation, and BAT, subWAT and liver were extracted from the mice. RNA was extracted from these tissues with TRIzol Reagent (Molecular Research Center, Inc., Cincinnati, OH, USA) following manufacturer’s instructions. RNA was isolated and quantified with a NanoDrop ND-2000 spectrophotometer (Thermo Fisher Scientific, Waltham, MA, USA). Total RNA isolated from BAT and subWAT was reverse transcribed, and quantitative real-time RT-PCR was performed as described previously [60,72]. The primers for quantitative real-time PCR are listed in Table 4.

### 4.5. Serum Chemistry

Blood was obtained from the mice, and serum glucose was measured with an Ascensia autoanalyzer (Bayer Medical, Ltd., Tokyo, Japan). Serum levels of NEFA, TG and TC were measured by enzymatic colorimetry with NEFA C, TG E and TC E test kits (Wako Pure Chemical Industries, Ltd., Osaka, Japan), respectively.

### 4.6. Western Blotting

To prepare tissue lysates, BAT and subWAT were homogenized on ice in ice-cold lysis buffer consisting of 25 mM Tris-HCl, pH 7.4, 10 mM sodium orthovanadate, 50 mM sodium pyrophosphate, 100 mM sodium fluoride, 10 mM EDTA, 10 mM EGTA, 1 mM phenylmethylsulfonyl fluoride and 1% NP-40 that supplemented with a protease inhibitor cocktail and phosphatase inhibitor cocktail (both, Roche Diagnostics, Mannheim, Germany). After centrifugation of the tissue homogenates at 14,000× *g* for 10 min at 4 °C, the supernatants were collected for determination of protein concentrations by Bradford protein assay using a Bio-Rad Protein Assay Kit (Bio-Rad Laboratories, Inc., Hercules, CA, USA). Proteins (5 μg for BAT or 25 μg for subWAT) were separated by SDS-PAGE (7.5% gel) and then transferred electrophoretically onto Clear Blot Membrane-P (ATTO, Tokyo, Japan) and immunoblotted with specific primary antibodies: UCP1 (ab10983, 1:2000 dilution; Abcam,) and β-actin (C4) (sc-47778, 1:5000 dilution; Santa Cruz Biotechnology, Inc., Santa Cruz, CA, USA). The secondary antibodies included goat anti-rabbit IgG (sc-2005, 1:8000 dilution) and m-IgGκ BP-HRP (sc-516102, 1:6000 dilution; both from Santa Cruz Biotechnology, Inc.). ECL detection reagents (Amersham Biosciences, Buckinghamshire, UK) were used to detect the desired proteins, which were then quantified with the NIH Image software program (NIH, Bethesda, MD, USA).

### 4.7. Statistical Analysis

Values are shown as the mean ± SEM. Significant differences between the mean values of the two groups were evaluated by Student *t*-test with IBM SPSS Statistics 23. Statistical significance was indicated by a *p* value < 0.05.

## Figures and Tables

**Table 1:** BW and weights of tissues in Con- and FO-fed mice.

BW/Tissues	Con-Fed	FO-Fed
BW at start (g)	23.1 ± 0.4	23.1 ± 0.2
Final BW (g)	25.9 ± 0.5	25.2 ± 0.4
BAT (g)	0.087 ± 0.006	0.083 ± 0.007
Subcutaneous WAT(g)	0.329 ± 0.029	0.251 ± 0.026
Epididymal WAT (g)	0.462 ± 0.041	0.326 ± 0.018 *
Mesenteric WAT (g)	0.149 ± 0.024	0.081 ± 0.008 *
Liver (g)	1.19 ± 0.03	1.22 ± 0.04

Values are mean ± SEM (n = 7). * *p* < 0.05 vs. Con-fed mice. Significant differences between two groups were tested by Student *t*-test. BW: body weight; Con: control; FO: fish oil; BAT: brown adipose tissue; WAT: white adipose tissue.

**Table 2:** Serum chemicals of Con- and FO-fed mice.

	Con-Fed	FO-Fed
Glucose (mg/dL)	169.6 ± 15.8	177.1 ± 11.4
NEFA (mEq/L)	0.83 ± 0.07	0.49 ± 0.06 **
TG (mg/dL)	179.9 ± 25.1	70.3 ± 16.4 **
TC (mg/dL)	179.0 ± 13.4	102.3 ± 3.7 ***

Values are mean ± SEM (n = 7). ** *p* < 0.01, *** *p* < 0.001 vs. Con-fed mice. Significant differences between two groups were tested by Student *t*-test. Con: control; FO: fish oil; NEFA: non-esterified fatty acid; TG: triglyceride; TC: total cholesterol.

**Table 3:** Dietary composition.

Dietary Constituents	Con	FO
	g/100 g	
Safflower oil (high oleic)	3.46	0.00
Butter	10.38	0.00
Fish oil	0.00	13.84
Casein	22.2	22.2
α-Starch	52.98	52.98
Vitamin mix (AIN-93)	1.12	1.12
Mineral mix (AIN-93)	3.92	3.92
Cellulose powder	5.60	5.60
L-Cystine	0.34	0.34
	en%	
Fat	30	30
Carbohydrate	50	50
Protein	20	20

Con: control; FO: fish oil; en%: energy %.

**Table 4:** Primers used for quantitative real-time PCR.

Gene	Forward Primer (5′ to 3′)	Reverse Primer (5′ to 3′)
*36b4*	GGCCCTGCACTCTCGCTTTC	TGCCAGGACGCGCTTGT
*Aco*	GCCCAACTGTGACTTCCATT	GGCATGTAACCCGTAGCACT
*β3-AR*	TCTAGTTCCCAGCGGAGTTTTCATCG	CGCGCACCTTCATAGCCATCAAACC
*Cidea*	ATCACAACTGGCCTGGTTACG	TACTACCCGGTGTCCATTTCT
*Cpt I*	GCACTGCAGCTCGCACATTACAA	CTCAGACAGTACCTCCTTCAGGAAA
*Dio2*	GCACGTCTCCAATCCTGAAT	TGAACCAAAGTTGACCACCA
*Fgf21*	ATGGAATGGATGAGATCTAGAGTTGG	TCTTGGTCGTCATCTGTGTAGAGG
*Mcad*	GATCGCAATGGGTGCTTTTGATAGAA	AGCTGATTGGCAATGTCTCCAGCAAA
*Pgc1α*	AAGTGTGGAACTCTCTGGAACTG	GGGTTATCTTGGTTGGCTTTATG
*Pparα*	CCTCAGGGTACCACTACGGAGT	GGTCTTCTTCTGAATCTTGCAGCT
*Prdm16*	GACATTCCAATCCCACCAGA	CACCTCTGTATCCGTCAGCA
*Ucp1*	GGCCCTTGTAAACAACAAAATAC	GGCAACAAGAGCTGACAGTAAAT

## Data Availability

The data presented in this study are included in the corresponding sections throughout the manuscript.

## Supplementary Materials

The following are available online at https://www.mdpi.com/article/10.3390/md19050278/s1, Table S1: Effect of fish oil (FO) supplementation on gene expression in liver.
