# Supplementary material for: Fish Oil Increases Diet-Induced Thermogenesis in Mice
Source: Mar Drugs. 2021 May 17;19(5):278. doi: 10.3390/md19050278 (PMC8156710; doi:10.3390/md19050278)
Supplement: Supplementary file 1 [file marinedrugs-19-00278-s001.zip › marinedrugs-1178739-supplementary.pdf]

Table S1. Effect of fish oil (FO) supplementation on gene expression in liver.

	Con	FO
Fatty acid β-oxidation		
Ppar α	100.0 ± 10.8	100.9 ± 10.8
Mcad	100.0 ± 9.0	181.3 ± 19.7*
CptI	100.0 ± 18.7	68.9 ± 7.3
Aco	100.0 ± 14.0	152.3 ± 21.5
Ucp2	100.0 ± 25.2	178.3 ± 45.0
Fatty acid synthesis		
Srebp-1c	100.0 ± 10.3	66.4 ± 12.8
Fas	100.0 ± 24.4	20.4 ± 4.8*
Scd1	100.0 ± 16.9	9.1 ± 2.5**
Acc1	100.0 ± 23.3	33.9 ± 8.2*

Values are mean ± SEM (n = 7). Con: control; FO: fish oil. *$p < 0.05$, **$p < 0.01$ vs. Con-fed mice.
